# Behavioural and social science research opportunities

**DOI:** 10.2471/BLT.20.285370

**Published:** 2021-08-31

**Authors:** Maria A Carrasco, Alexandria K Mickler, Ruth Young, Kaitlyn Atkins, Joseph G Rosen, Rafael Obregon

**Affiliations:** a United States Agency for International Development (USAID), Office of Population and Reproductive Health; 500 D Street SW, 05.4.1A, Washington, DC, United States of America (USA).; b Johns Hopkins Bloomberg School of Public Health, Department of International Health, Baltimore, USA.; c United Nations Children’s Fund, Asuncion, Paraguay.

Incorporating behavioural insights into health policies, interventions and systems has helped reduce injury-related mortality, improve adherence to medications and reduce tobacco use.^1^ Nevertheless, health practitioners and policy-makers sometimes overlook behaviourally informed and focused approaches. For instance, early coronavirus disease 2019 (COVID-19) prevention efforts in the United States of America relied on best-case modelling scenarios, which assumed widespread adoption of preventive behaviours like face-mask use. Despite compulsory face-mask mandates, behavioural adoption was slow; once vaccines were available, officials then focused on vaccine uptake. Adequate vaccine uptake, in turn, depends on incorporating behavioural insights to address vaccine hesitancy. Indeed, vaccine administration, and not vaccines alone, is needed to help curb the COVID-19 pandemic. Voluntary vaccine uptake requires creating an enabling environment based on trust, working with social influencers and respected opinion leaders to model vaccine uptake, and providing appropriate motivation such as vaccine passports that facilitate travel, among other actions. While incorporation of behavioural insights into health policies, interventions and systems is gaining momentum, challenges remain. Here we describe three challenges in behavioural and social science research that hamper the integration of behavioural insights and we highlight opportunities for addressing them.

## Methodological challenges

Social and behavioural issues are complex and adaptive, and fully understanding their impact requires the use of similarly dynamic, multidimensional approaches. For example, random assignment is a unique challenge for social behaviour change trials, particularly for national media-based interventions where random assignment to intervention arms is infeasible. This difficulty leads researchers to turn to more complex study designs and statistical approaches to provide unbiased estimates of treatment effects.^2^ However, such approaches are resource-intensive, and ensuring their appropriate interpretation down the research pipeline can be challenging.

Behavioural science researchers also face the challenge of measuring key psychosocial, contextual and structural factors that influence health. While progress has been made, existing measures of these factors require constant adaptation and refinement based on context. Furthermore, many health behaviour measures are self-reported and subject to social desirability and recall biases. List experimentation techniques and negatively framed questions in one recent population-based survey, for example, were shown to significantly reduce self-reported compliance with recommended public health measures during the COVID-19 pandemic.^3^ Where possible, behavioural studies should integrate additional, more objective indicators (for example biomarkers, attendance records and health clinic registers) and apply techniques to minimize bias in self-reported data, such as self-interviewing and unmatched counting. Similarly, indicators used to assess social behaviour change programme coverage and impact, such as number of media communications received and condom use at last sex, are often unstable, that is, subject to change easily with small environmental adjustments or bias. Recognizing these indicators’ weaknesses can ensure appropriate interpretation of results and the potential development of more stable measures. Elicitation techniques like media recall items, where survey respondents are asked to finish a slogan from a mass media communication, are more nuanced measures for appraising social behaviour change communication intervention coverage. Assessing programme impact is further complicated by the scarcity of modelling studies linking social behaviour change interventions with impact measures such as number of deaths averted. Modelling can provide important information related to the population-level impact of behaviourally informed or focused investments to ensure equitable resource allocation and support advocacy efforts.

Despite these challenges, rigorous behavioural research and evaluations in non-controlled settings are ongoing. Ecological momentary assessments have shown promise for capturing psychosocial, behavioural and intervention outcomes using real-time data capture.^4^ Natural experiments have provided causal evidence around the impact of mass media interventions on fertility.^5^ Experimental research in mass media and communications has identified intervention effects by comparing outcomes among listener groups who received targeted social and behaviour mass media campaigns, compared to controls who received typical mass media messages.^6^^,^^7^ Donors and peer-reviewed journals should support the use and development of these and other promising new methods for data analysis through funding and publication opportunities, even in cases of null findings.

## Limited data availability and use

The integration of behavioural insights into interventions and health policies has been hampered by a lack of data availability and use. Many behavioural science studies are not designed nor used to their maximum potential. Full descriptions of interventions, curriculums or protocols are typically not readily available for replication, thereby reducing opportunities for standardization across programmes and contexts. Similarly, no systematic reporting of costing or cost–effectiveness data exists, preventing cost comparisons and complicating the ability to determine scale-up or replication costs. Limited availability and use of data are also a barrier to determining and analysing opportunities for improved impact in cases where an intervention did not achieve intended results.

While qualitative and quantitative data from behavioural science studies could be used to conduct secondary analyses, these analyses are rare because such data are not openly shared. Oftentimes, systems are not in place to share data with interested stakeholders and researchers in a way that protects the anonymity of research participants. However, making data available is critical for transparency and accountability. Furthermore, widely accessible data can enable local researchers to include indigenous perspectives in addressing local concerns and providing opportunities for knowledge sharing and strengthened data analysis skills, as well as enhancing the presentation and utilization of evidence. In this area, international donors can have an important impact by requiring researchers to make protocols, data collection instruments and de-identified data (that is, that cannot be traced to the study participant) publicly available in a timely manner. Donors can also invest in online data-sharing platforms that outlive project lifecycles. Additionally, international donors and multilateral organizations should encourage research collaboration with local researchers and fund local data analysis and capacity-strengthening activities.

## Key gaps

Researchers, policy-makers and practitioners are often unable to cite evidence-based strategies promoting behaviour change and leading to improved health outcomes. Part of the challenge rests with researchers’ tendency to seek simple intervention main effects when the more informative analytical approach would be to identify factors in the pathway between behavioural approaches and health outcomes. In other words, evaluations tend to ask whether interventions achieve desired outcomes, without focusing on explaining why and how these interventions work or not. Additionally, interventions do not affect everyone uniformly; rather, some people are affected under some conditions but not others. Therefore, the contextual factors affecting the intervention’s impact must also be considered. Furthermore, researchers do not typically unpack the contribution of each behavioural strategy employed in multicomponent interventions to measured health outcomes. A recent analysis of behavioural interventions in family planning, for example, aggregated multicomponent interventions into a packages category and estimated their effects on modern contraceptive uptake, since the aggregated studies were not designed to provide individual component effects.^8^ To build a strong evidence base and develop strategies for translating behavioural insights across contexts, studies guided by theories of change that examine psychosocial pathways and moderators are key to building the evidence base and strategies for translating behavioural insights across contexts. Funders should prioritize these types of studies.

While the number of high-quality evaluations testing behavioural pathways has grown in the last decade, their dissemination in practitioner and policy-maker circles remains limited. Delegates attending the first International Social and Behaviour Change Communication Summit in Addis Ababa (2016) reached similar conclusions.^9^ Furthermore, a stakeholder analysis to inform this field for adolescent sexual and reproductive health programmes highlights that practitioners experience challenges integrating science into practice and that general guidance, tools and strategy standardization are needed.^10^ Thus, enhanced research utilization efforts are needed.

The High Impact Practices in Family Planning, an authoritative evidence synthesis, summarize some of the social and behaviour change literature in succinct briefs, using nontechnical language, for decision-makers and implementers.^11^ However, the High-Impact Practices Technical Advisory Group has recognized that the current briefs, focused on channels of communication, are too broad to inform family planning investments. Thus, new high impact practices briefs are being developed with evidence syntheses on strategies for advancing intermediate outcomes, such as couples communication, social norms, beliefs and attitudes associated with various family planning outcomes (for example, achieving fertility intentions, full and informed method choice and modern contraceptive uptake). Another example focusing on analysing pathways to change is the work completed under the ACCELERATE project, which identified priority behaviours (or intermediate outcomes) in the pathway leading to ultimate desired health outcomes in maternal and child health. The ThinkBig website, initially developed by ACCELERATE, offers numerous resources for practitioners to integrate behavioural insights into public health programmes.^12^ The High Impact Practices and ACCELERATE are examples of strategies to facilitate behavioural insights integration and knowledge utilization, by synthesizing evidence using accessible language that explains complex behavioural pathways. Practitioners and policy-makers should use these and similar tools to integrate behavioural and social science evidence into public health policies and programmes. Donors should prioritize investments to help translate behavioural and social science evidence into practice by integrating research utilization into programme and research design.
